# Supplementary material for: Drug therapy and other factors associated with the development of acute kidney injury in critically ill patients: a cross-sectional study
Source: PeerJ. 2018 Aug 14;6:e5405. doi: 10.7717/peerj.5405 (PMC6097492; doi:10.7717/peerj.5405)
Supplement: Supplemental Information 1 [file peerj-06-5405-s001.pdf]

| ctrl | codpesq | idade | sexo | corpele | setorig | histetil | tabagis | apache_ii | cid10 | comorb | iam | icc |
|---|---|---|---|---|---|---|---|---|---|---|---|---|
| 1 | 1 | 47 | 1 | 4 | 2 | 2 | 2 | | AVC NAO ESPECI | 2 | 1 | 1 |
| 2 | 2 | 34 | 1 | 4 | 3 | 1 | 1 | | QUEIMADURA D | 1 | 1 | 1 |
| 3 | 3 | 86 | 1 | 1 | 1 | 1 | 1 | | INSUFICIENCIA R | 2 | 1 | 1 |
| 4 | 4 | 18 | 2 | 2 | 3 | 1 | 2 | 42 | TRAUMATISMOS | 1 | 1 | 1 |
| 5 | 5 | 30 | 2 | 3 | 3 | 1 | 1 | | TRAUMATISMOS | 1 | 1 | 1 |
| 6 | 6 | 68 | 2 | 4 | 3 | 1 | 1 | | AVC NAO ESPECI | 2 | 1 | 1 |
| 7 | 7 | 58 | 2 | 2 | 3 | 2 | 1 | | DISPNEIA | 1 | 1 | 1 |
| 8 | 8 | 28 | 2 | 4 | 3 | 1 | 1 | | FRATURA DO AC | 1 | 1 | 1 |
| 9 | 9 | 60 | 2 | 4 | 1 | 1 | 1 | | INSUFICIENCIA C | 2 | 1 | 2 |
| 11 | 11 | 24 | 1 | 3 | 1 | 1 | 1 | | GASTRITE NAO E | 2 | 1 | 1 |
| 12 | 12 | 55 | 2 | 3 | 1 | 2 | 1 | 45 | HEMATEMESE | 2 | 1 | 1 |
| 13 | 13 | 55 | 1 | 2 | 1 | 1 | 1 | | ASMA NAO ESPE | 2 | 1 | 1 |
| 14 | 14 | 53 | 1 | 4 | 1 | 8 | 8 | | HEMORRAGIA IN | 1 | 1 | 1 |
| 15 | 15 | 35 | 2 | 3 | 1 | 8 | 8 | | DM INSULINO DI | 2 | 1 | 1 |
| 16 | 16 | 75 | 1 | 4 | 1 | 1 | 1 | | ICC | 2 | 1 | 2 |
| 17 | 17 | 83 | 1 | 2 | 3 | 1 | 1 | | ATEROSCLEROSE | 2 | 2 | 2 |
| 18 | 18 | 19 | 2 | 4 | 1 | 2 | 2 | | TRAUMATISMO | 1 | 1 | 1 |
| 19 | 19 | 45 | 2 | 1 | 1 | 1 | 1 | | EPILEPSIA NAO E | 2 | 1 | 1 |
| 20 | 20 | 62 | 2 | 5 | 3 | 2 | 1 | | DOENCAS VASCU | 2 | 2 | 1 |
| 23 | 23 | 55 | 2 | 4 | 3 | 2 | 2 | 45 | HEMORRAGIA SU | 1 | 1 | 1 |
| 24 | 24 | 70 | 2 | 4 | 1 | 2 | 1 | 47 | AVC NAO ESPECI | 2 | 1 | 2 |
| 25 | 25 | 39 | 2 | 2 | 1 | 2 | 1 | | ENFORCAMENTO | 1 | 1 | 1 |
| 26 | 26 | 24 | 2 | 3 | 1 | 2 | 2 | | TRAUMATISMOS | 1 | 1 | 1 |
| 27 | 27 | 55 | 2 | 1 | 1 | 2 | 2 | | PARADA CARDIA | 2 | 2 | 2 |
| 28 | 28 | 61 | 1 | 1 | 1 | 1 | 1 | | AVC NAO ESPECI | 2 | 1 | 1 |
| 29 | 29 | 26 | 2 | 3 | 3 | 2 | 2 | | HEMORRAGIA EX | 1 | 1 | 1 |
| 30 | 30 | 27 | 1 | 1 | 1 | 1 | 2 | | TRANSTORNOS M | 2 | 1 | 1 |
| 31 | 31 | 39 | 1 | 4 | 3 | 1 | 1 | | FISTULA DO INTE | 1 | 1 | 1 |
| 32 | 32 | 84 | 2 | 2 | 1 | 2 | 1 | | DOENCA DO APA | 2 | 1 | 1 |
| 33 | 33 | 45 | 2 | 2 | 3 | 2 | 1 | 16 | AVC NAO ESPECI | 1 | 1 | 1 |

| | | | | | | | | | | | | |
|---|---|---|---|---|---|---|---|---|---|---|---|---|
| 34 | 34 | 83 | 1 | 1 | 1 | 1 | 1 | | AVC NAO ESPEC | 2 | 1 | 1 |
| 35 | 35 | 27 | 2 | 4 | 1 | 8 | 8 | | HEMOPTISE | 1 | 1 | 1 |
| 36 | 36 | 25 | 2 | 4 | 3 | 2 | 2 | | TRAUMATISMO | 2 | 1 | 1 |
| 37 | 37 | 77 | 1 | 4 | 3 | 1 | 1 | | ATEROSCLEROS | 2 | 1 | 1 |
| 38 | 38 | 83 | 1 | 4 | 1 | 1 | 1 | | INSUFICIENCIA R | 2 | 1 | 1 |
| 39 | 39 | 19 | 1 | 4 | 1 | 1 | 1 | 14 | DM INSULINO D | 2 | 1 | 1 |
| 40 | 40 | 39 | 2 | 3 | 3 | 1 | 1 | 14 | FISTULA DO INT | 2 | 1 | 1 |
| 41 | 41 | 54 | 2 | 1 | 3 | 1 | 1 | | EMBOLIA E TRO | 2 | 1 | 1 |
| 42 | 42 | 34 | 2 | 4 | 3 | 1 | 1 | 19 | ABDOME AGUD | 2 | 1 | 1 |
| 43 | 43 | 74 | 2 | 4 | 1 | 1 | 1 | 13 | CONCUSSAO CE | 2 | 2 | 2 |
| 44 | 44 | 22 | 1 | 4 | 1 | 2 | 2 | 16 | EFEITO TOXICO | 1 | 1 | 1 |
| 45 | 45 | 28 | 2 | 4 | 3 | 1 | 1 | | RUPTURA DE AR | 1 | 1 | 1 |
| 46 | 46 | 81 | 2 | 2 | 3 | 2 | 1 | 16 | ATEROSCLEROS | 2 | 1 | 1 |
| 47 | 47 | 37 | 1 | 2 | 2 | 1 | 1 | 8 | PARTO ESPONT | 2 | 1 | 1 |
| 48 | 48 | 46 | 1 | 4 | 1 | 1 | 1 | 25 | SINDROME DO | 2 | 1 | 1 |
| 49 | 49 | 35 | 2 | 4 | 1 | 2 | 2 | 9 | PNEUMONIA BA | 1 | 1 | 1 |
| 50 | 50 | 51 | 2 | 4 | 2 | 1 | 1 | 11 | HEMOPNEUMO | 2 | 1 | 1 |
| 51 | 51 | 41 | 2 | 4 | 3 | 1 | 1 | 42 | TRAUMATISMO | 1 | 1 | 1 |
| 52 | 52 | 87 | 1 | 4 | 3 | 1 | 1 | 36 | CONSTIPACAO | 2 | 1 | 2 |
| 53 | 53 | 53 | 1 | 1 | 3 | 2 | 2 | 15 | EMBOLIA E TRO | 2 | 1 | 1 |
| 54 | 54 | 41 | 1 | 4 | 3 | 2 | 1 | 20 | FERIMENTOS M | 2 | 1 | 1 |
| 55 | 55 | 46 | 1 | 1 | 1 | 2 | 2 | 40 | OUTRAS PNEUM | 2 | 1 | 1 |
| 56 | 56 | 18 | 2 | 2 | 3 | 1 | 1 | 31 | TRAUMATISMO | 1 | 1 | 1 |
| 57 | 57 | 78 | 2 | 3 | 1 | 1 | 1 | 25 | HEMORRAGIA I | 1 | 1 | 1 |
| 58 | 58 | 18 | 2 | 4 | 1 | 1 | 1 | 19 | EPILEPSIA NAO | 1 | 1 | 1 |
| 59 | 59 | 63 | 2 | 2 | 3 | 1 | 1 | | AVC | 2 | 2 | 1 |
| 60 | 60 | 69 | 2 | 4 | 3 | 2 | 1 | | AVC NAO ESPEC | 2 | 1 | 1 |
| 61 | 61 | 44 | 2 | 4 | 3 | 2 | 1 | | EMBOLIA E TRO | 1 | 1 | 1 |
| 62 | 62 | 74 | 1 | 1 | 3 | 1 | 1 | | FRATURA PERTR | 2 | 1 | 1 |
| 63 | 63 | 73 | 1 | 4 | 3 | 1 | 1 | 18 | GANGRENA NA | 2 | 1 | 1 |
| 64 | 64 | 32 | 2 | 2 | 3 | 2 | 2 | 20 | FRATURA DE M | 1 | 1 | 1 |

| | | | | | | | | | | | | |
|---|---|---|---|---|---|---|---|---|---|---|---|---|
| 65 | 65 | 41 | 1 | 4 | 3 | 1 | 1 | 8 | FRATURA DA EXT | 1 | 1 | 1 |
| 66 | 66 | 39 | 2 | 1 | 1 | 1 | 1 | 17 | TRAUMATISMO | 1 | 1 | 1 |
| 67 | 67 | 75 | 1 | 4 | 3 | 2 | 2 | 15 | EMBOLIA E TROI | 1 | 1 | 1 |
| 68 | 68 | 18 | 2 | 4 | 3 | 2 | 2 | 24 | TRAUMATISMOS | 2 | 1 | 1 |
| 69 | 69 | 80 | 1 | 4 | 3 | 1 | 2 | 27 | OUTRAS OBSTRU | 2 | 1 | 1 |
| 70 | 70 | 47 | 2 | 4 | 2 | 2 | 1 | 9 | GANGRENA NAC | 2 | 1 | 1 |
| 71 | 71 | 60 | 2 | 1 | 1 | 1 | 2 | 24 | SEPSE FOCO PUL | 2 | 1 | 1 |
| 72 | 72 | 28 | 1 | 1 | 2 | 1 | 1 | 9 | GRAVIDEZ DUPL | 1 | 1 | 1 |
| 73 | 73 | 35 | 2 | 4 | 3 | 2 | 2 | 22 | TRAUMATISMOS | 2 | 1 | 2 |
| 74 | 74 | 48 | 2 | 4 | 1 | 2 | 1 | | PNEUMONIA BA | 2 | 1 | 2 |
| 75 | 75 | 66 | 2 | 1 | 1 | 1 | 2 | 24 | FRATURA DE OU | 1 | 1 | 1 |
| 76 | 76 | 28 | 2 | 2 | 3 | 1 | 1 | 20 | FRATURA DA EXT | 1 | 1 | 1 |
| 77 | 77 | 24 | 2 | 4 | 3 | 2 | 2 | 13 | TRAUMATISMOS | 1 | 1 | 1 |
| 78 | 78 | 36 | 2 | 1 | 3 | 1 | 1 | 5 | TRAUMATISMOS | 1 | 1 | 1 |
| 79 | 79 | 81 | 1 | 2 | 2 | 1 | 1 | 25 | AVC | 2 | 1 | 1 |
| 80 | 80 | 31 | 2 | 2 | 3 | 2 | 2 | 15 | FRATURA DA AB | 1 | 1 | 1 |
| 81 | 81 | 64 | 2 | 4 | 3 | 2 | 2 | 11 | GANGRENA NAC | 2 | 2 | 2 |
| 82 | 82 | 37 | 2 | 1 | 3 | 1 | 1 | | TRAUMATISMOS | 2 | 1 | 1 |
| 83 | 83 | 46 | 2 | 4 | 1 | 1 | 1 | 24 | INSUFICIENCIA R | 2 | 1 | 1 |
| 84 | 84 | 26 | 1 | 2 | 3 | 1 | 1 | 22 | TRAUMATISMOS | 1 | 1 | 1 |
| 85 | 85 | 54 | 1 | 4 | 3 | 1 | 1 | | CALCULOSE DE V | 1 | 1 | 1 |
| 86 | 86 | 79 | 1 | 4 | 3 | 1 | 1 | 20 | ATEROSCLEROSE | 2 | 1 | 1 |
| 87 | 87 | 77 | 1 | 1 | 3 | 1 | 1 | 26 | AVC NAO ESPECI | 2 | 1 | 1 |
| 88 | 88 | 86 | 1 | 1 | 2 | 1 | 1 | 17 | FRATURA SUBTR | 2 | 1 | 1 |
| 89 | 89 | 38 | 2 | 3 | 1 | 2 | 2 | 18 | EPILEPSIA NAO E | 1 | 1 | 1 |
| 90 | 90 | 76 | 2 | 1 | 1 | 2 | 1 | 29 | MIOCARDIOPATI | 2 | 1 | 1 |
| 91 | 91 | 81 | 2 | 1 | 1 | 1 | 1 | | ANGINA PECTOR | 1 | 1 | 1 |
| 92 | 92 | 19 | 2 | 1 | 3 | 1 | 1 | 12 | TRAUMATISMOS | 2 | 1 | 1 |
| 93 | 93 | 34 | 1 | 4 | 1 | 1 | 1 | 18 | EFEITO TOXICO D | 1 | 1 | 1 |
| 94 | 94 | 21 | 1 | 4 | 2 | 1 | 1 | 13 | PARTO ESPONTA | 2 | 1 | 1 |
| 95 | 95 | 56 | 2 | 1 | 3 | 2 | 2 | | APENDICITE AGU | 2 | 2 | 1 |

| | | | | | | | | | | | | |
|---|---|---|---|---|---|---|---|---|---|---|---|---|
| 96 | 96 | 46 | 1 | 3 | 1 | 2 | 1 | 28 | EPILEPSIA E SIND | 2 | 1 | 1 |
| 97 | 97 | 88 | 1 | 1 | 1 | 1 | 1 | 18 | PNEUMONIA BA | 2 | 1 | 1 |
| 98 | 98 | 21 | 2 | 2 | 3 | 2 | 2 | 18 | TRAUMATISMOS | 1 | 1 | 1 |
| 99 | 99 | 29 | 2 | 4 | 1 | 1 | 2 | | ENCEFALITE VIRA | 1 | 1 | 1 |
| 100 | 100 | 34 | 2 | 4 | 3 | 2 | 2 | 20 | TRAUMATISMOS | 1 | 1 | 1 |
| 101 | 101 | 25 | 2 | 2 | 3 | 2 | 2 | | TRAUMATISMOS | 1 | 1 | 1 |
| 102 | 102 | 85 | 1 | 4 | 1 | 1 | 1 | 28 | AVC NAO ESPECI | 1 | 1 | 2 |
| 103 | 103 | 63 | 1 | 1 | 1 | 2 | 1 | | SEPTICEMIA NAO | 1 | 1 | 1 |
| 104 | 104 | 20 | 2 | 4 | 3 | 1 | 1 | 17 | SEQUELAS DE HE | 1 | 1 | 1 |
| 105 | 105 | 20 | 2 | 4 | 3 | 1 | 1 | 23 | TRAUMATISMOS | 1 | 1 | 1 |
| 106 | 106 | 53 | 1 | 1 | 1 | 1 | 1 | 10 | EFEITO TOXICO D | 1 | 1 | 1 |
| 107 | 107 | 27 | 1 | 4 | 2 | 1 | 1 | 15 | GRAVIDEZ ABDO | 1 | 1 | 1 |
| 108 | 108 | 41 | 2 | 2 | 3 | 2 | 1 | 7 | ABSCESSO E GRA | 1 | 1 | 1 |
| 109 | 109 | 69 | 1 | 4 | 1 | 1 | 1 | 23 | AVC NAO ESPECI | 2 | 1 | 1 |
| 110 | 110 | 60 | 2 | 2 | 2 | 2 | 2 | | AVC NAO ESPECI | 1 | 2 | 2 |
| 111 | 111 | 70 | 2 | 4 | 1 | 1 | 1 | 30 | PARADA CARDIA | 1 | 1 | 2 |
| 112 | 112 | 74 | 2 | 4 | 3 | 1 | 1 | 10 | OUTRAS FORMA | 1 | 1 | 1 |
| 113 | 113 | 75 | 1 | 4 | 1 | 1 | 1 | 31 | AVC | 1 | 2 | 2 |
| 114 | 114 | 35 | 2 | 2 | 1 | 1 | 1 | | EPILEPSIA NAO E | 1 | 1 | 1 |
| 115 | 115 | 26 | 1 | 2 | 2 | 1 | 2 | 13 | GRAVIDEZ TUBA | 1 | 1 | 1 |
| 116 | 116 | 41 | 1 | 4 | 3 | 1 | 2 | | OUTRAS DOENCA | 1 | 1 | 1 |
| 117 | 117 | 75 | 1 | 4 | 2 | 1 | 1 | 2 | FRATURA PERTR | 1 | 1 | 1 |
| 118 | 118 | 82 | 2 | 1 | 1 | 2 | 1 | 27 | ANURIA E OLIGU | 2 | 1 | 1 |
| 119 | 119 | 39 | 1 | 4 | 1 | 1 | 2 | 13 | HIPERTENSAO ES | 1 | 1 | 1 |
| 120 | 120 | 19 | 2 | 4 | 3 | 2 | 2 | 22 | FERIMENTOS MU | 1 | 1 | 1 |
| 121 | 121 | 52 | 2 | 4 | 3 | 1 | 1 | 24 | HEMOTORAX | 1 | 1 | 1 |
| 122 | 122 | 24 | 2 | 4 | 1 | 2 | 1 | 15 | PARADA CARDIA | 1 | 1 | 1 |
| 123 | 123 | 82 | 1 | 1 | 1 | 1 | 2 | | OUTROS SINTOM | 2 | 1 | 2 |
| 124 | 124 | 24 | 1 | 4 | 2 | 1 | 1 | | PARTO ESPONTA | 1 | 1 | 1 |
| 125 | 125 | 68 | 1 | 4 | 1 | 2 | 1 | | AVC | 1 | 1 | 1 |

| dvp | dcervasc | demencia | dpulmona | dconjunt | ulcera | hepleve | dhepseve | diabetes | diabcomp | hemipleg | drenal | neoplas | leucem |
|---|---|---|---|---|---|---|---|---|---|---|---|---|---|
| 1 | 1 | 1 | 2 | 1 | 1 | 1 | 1 | 1 | 1 | 1 | 1 | 1 | 1 |
| 1 | 1 | 1 | 1 | 1 | 1 | 1 | 1 | 1 | 1 | 1 | 1 | 1 | 1 |
| 1 | 1 | 2 | 1 | 1 | 2 | 1 | 1 | 2 | 1 | 2 | 1 | 1 | 1 |
| 1 | 1 | 1 | 1 | 1 | 1 | 1 | 1 | 1 | 1 | 1 | 1 | 1 | 1 |
| 1 | 1 | 1 | 1 | 1 | 1 | 1 | 1 | 1 | 1 | 1 | 1 | 1 | 1 |
| 2 | 2 | 1 | 1 | 1 | 1 | 1 | 1 | 2 | 2 | 1 | 1 | 1 | 1 |
| 1 | 1 | 1 | 1 | 1 | 1 | 1 | 1 | 1 | 1 | 1 | 1 | 1 | 1 |
| 1 | 1 | 1 | 1 | 1 | 1 | 1 | 1 | 1 | 1 | 1 | 1 | 1 | 1 |
| 1 | 1 | 1 | 1 | 1 | 1 | 1 | 1 | 1 | 1 | 2 | 1 | 1 | 1 |
| 1 | 1 | 1 | 1 | 1 | 1 | 1 | 1 | 2 | 2 | 1 | 1 | 1 | 1 |
| 1 | 1 | 1 | 1 | 1 | 1 | 1 | 2 | 1 | 1 | 1 | 1 | 1 | 1 |
| 1 | 1 | 1 | 2 | 2 | 1 | 1 | 1 | 1 | 1 | 1 | 1 | 1 | 1 |
| 1 | 1 | 1 | 1 | 1 | 1 | 1 | 1 | 1 | 1 | 1 | 1 | 1 | 1 |
| 1 | 1 | 1 | 1 | 1 | 2 | 1 | 1 | 2 | 1 | 1 | 1 | 1 | 1 |
| 2 | 1 | 1 | 2 | 2 | 1 | 1 | 1 | 1 | 1 | 1 | 1 | 1 | 1 |
| 1 | 1 | 1 | 1 | 1 | 1 | 1 | 1 | 2 | 2 | 1 | 1 | 1 | 1 |
| 1 | 1 | 1 | 1 | 1 | 1 | 1 | 1 | 1 | 1 | 1 | 1 | 1 | 1 |
| 1 | 1 | 1 | 1 | 1 | 1 | 1 | 1 | 1 | 1 | 2 | 1 | 1 | 1 |
| 2 | 1 | 1 | 1 | 1 | 1 | 1 | 1 | 1 | 1 | 1 | 1 | 1 | 1 |
| 1 | 1 | 1 | 1 | 1 | 1 | 1 | 1 | 1 | 1 | 1 | 1 | 1 | 1 |
| 1 | 2 | 1 | 2 | 1 | 1 | 1 | 1 | 1 | 1 | 2 | 1 | 1 | 1 |
| 1 | 1 | 1 | 1 | 1 | 1 | 1 | 1 | 1 | 1 | 1 | 1 | 1 | 1 |
| 1 | 1 | 1 | 1 | 1 | 1 | 1 | 1 | 1 | 1 | 1 | 1 | 1 | 1 |
| 1 | 1 | 1 | 1 | 1 | 1 | 1 | 1 | 1 | 1 | 1 | 1 | 1 | 1 |
| 1 | 2 | 1 | 1 | 1 | 2 | 1 | 1 | 1 | 1 | 1 | 1 | 1 | 1 |
| 1 | 1 | 1 | 1 | 1 | 1 | 1 | 1 | 1 | 1 | 1 | 1 | 1 | 1 |
| 1 | 1 | 1 | 2 | 1 | 1 | 1 | 1 | 1 | 1 | 1 | 1 | 1 | 1 |
| 1 | 1 | 1 | 1 | 1 | 1 | 1 | 1 | 1 | 1 | 1 | 1 | 1 | 1 |
| 1 | 2 | 1 | 1 | 1 | 1 | 1 | 1 | 1 | 1 | 1 | 1 | 1 | 1 |
| 1 | 1 | 1 | 1 | 1 | 1 | 1 | 1 | 1 | 1 | 1 | 1 | 1 | 1 |

| | | | | | | | | | | | | | |
|---|---|---|---|---|---|---|---|---|---|---|---|---|---|
| 1 | 2 | 1 | 1 | 2 | 1 | 2 | 1 | 1 | 1 | 1 | 1 | 1 | 1 |
| 1 | 1 | 1 | 1 | 1 | 1 | 1 | 1 | 1 | 1 | 1 | 1 | 1 | 1 |
| 1 | 1 | 1 | 1 | 1 | 1 | 1 | 1 | 1 | 1 | 2 | 1 | 1 | 1 |
| 2 | 1 | 1 | 2 | 2 | 1 | 1 | 1 | 1 | 1 | 1 | 1 | 1 | 1 |
| 1 | 1 | 1 | 2 | 1 | 1 | 1 | 1 | 1 | 1 | 1 | 1 | 1 | 1 |
| 1 | 1 | 1 | 1 | 1 | 1 | 1 | 1 | 2 | 1 | 1 | 1 | 1 | 1 |
| 1 | 1 | 1 | 1 | 1 | 2 | 1 | 1 | 1 | 1 | 1 | 1 | 1 | 1 |
| 1 | 2 | 1 | 1 | 1 | 1 | 1 | 1 | 2 | 1 | 2 | 1 | 1 | 1 |
| 1 | 1 | 1 | 2 | 2 | 1 | 1 | 1 | 1 | 1 | 1 | 1 | 1 | 1 |
| 1 | 1 | 1 | 1 | 1 | 1 | 1 | 1 | 1 | 1 | 1 | 1 | 1 | 1 |
| 1 | 1 | 1 | 1 | 1 | 1 | 1 | 1 | 1 | 1 | 1 | 1 | 1 | 1 |
| 1 | 1 | 1 | 1 | 1 | 1 | 1 | 1 | 1 | 1 | 1 | 1 | 1 | 1 |
| 1 | 2 | 1 | 1 | 1 | 2 | 1 | 1 | 1 | 1 | 1 | 1 | 1 | 1 |
| 1 | 1 | 1 | 1 | 1 | 1 | 1 | 1 | 2 | 1 | 1 | 1 | 1 | 1 |
| 2 | 1 | 1 | 2 | 1 | 1 | 1 | 1 | 1 | 1 | 1 | 1 | 1 | 1 |
| 1 | 1 | 1 | 1 | 1 | 1 | 1 | 1 | 1 | 1 | 1 | 1 | 1 | 1 |
| 1 | 1 | 1 | 2 | 1 | 1 | 1 | 1 | 1 | 1 | 1 | 1 | 1 | 1 |
| 1 | 1 | 1 | 1 | 1 | 1 | 1 | 1 | 1 | 1 | 1 | 1 | 1 | 1 |
| 1 | 2 | 1 | 1 | 1 | 1 | 1 | 1 | 2 | 1 | 1 | 1 | 1 | 1 |
| 1 | 1 | 1 | 1 | 1 | 2 | 1 | 1 | 2 | 2 | 1 | 1 | 1 | 1 |
| 1 | 1 | 1 | 2 | 2 | 1 | 1 | 2 | 1 | 1 | 1 | 1 | 1 | 1 |
| 2 | 1 | 1 | 1 | 1 | 1 | 1 | 1 | 1 | 1 | 1 | 1 | 1 | 1 |
| 1 | 1 | 1 | 1 | 1 | 1 | 1 | 1 | 1 | 1 | 1 | 1 | 1 | 1 |
| 1 | 1 | 1 | 1 | 1 | 1 | 1 | 1 | 1 | 1 | 1 | 1 | 1 | 1 |
| 1 | 1 | 1 | 1 | 1 | 1 | 1 | 1 | 1 | 1 | 1 | 1 | 1 | 1 |
| 1 | 2 | 1 | 1 | 1 | 1 | 1 | 1 | 2 | 1 | 1 | 1 | 1 | 1 |
| 2 | 2 | 1 | 1 | 2 | 2 | 1 | 1 | 1 | 1 | 1 | 2 | 1 | 1 |
| 1 | 1 | 1 | 1 | 1 | 1 | 1 | 1 | 1 | 1 | 1 | 1 | 1 | 1 |
| 1 | 2 | 1 | 1 | 1 | 1 | 1 | 1 | 1 | 1 | 1 | 1 | 1 | 1 |
| 2 | 1 | 1 | 1 | 1 | 2 | 1 | 1 | 2 | 2 | 1 | 1 | 1 | 1 |
| 1 | 1 | 1 | 1 | 1 | 1 | 1 | 1 | 1 | 1 | 1 | 1 | 1 | 1 |

| | | | | | | | | | | | | | |
|---|---|---|---|---|---|---|---|---|---|---|---|---|---|
| 1 | 1 | 1 | 1 | 1 | 1 | 1 | 1 | 1 | 1 | 1 | 1 | 1 | 1 |
| 1 | 1 | 1 | 1 | 1 | 1 | 1 | 1 | 1 | 1 | 1 | 1 | 1 | 1 |
| 1 | 1 | 1 | 1 | 1 | 1 | 1 | 1 | 1 | 1 | 1 | 1 | 1 | 1 |
| 1 | 2 | 1 | 1 | 1 | 1 | 1 | 1 | 1 | 1 | 1 | 1 | 1 | 1 |
| 1 | 1 | 1 | 1 | 1 | 1 | 1 | 1 | 2 | 1 | 1 | 1 | 2 | 1 |
| 1 | 1 | 1 | 1 | 1 | 1 | 1 | 1 | 2 | 1 | 1 | 1 | 1 | 1 |
| 1 | 1 | 1 | 2 | 1 | 1 | 1 | 1 | 2 | 2 | 1 | 2 | 1 | 1 |
| 1 | 1 | 1 | 1 | 1 | 1 | 1 | 1 | 1 | 1 | 1 | 1 | 1 | 1 |
| 2 | 1 | 1 | 2 | 1 | 1 | 1 | 1 | 1 | 1 | 1 | 1 | 1 | 1 |
| 1 | 1 | 1 | 2 | 2 | 1 | 1 | 1 | 1 | 1 | 1 | 1 | 1 | 1 |
| 1 | 1 | 1 | 1 | 1 | 1 | 1 | 1 | 1 | 1 | 1 | 1 | 1 | 1 |
| 1 | 1 | 1 | 1 | 1 | 1 | 1 | 1 | 1 | 1 | 1 | 1 | 1 | 1 |
| 1 | 1 | 1 | 1 | 1 | 1 | 1 | 1 | 1 | 1 | 1 | 1 | 1 | 1 |
| 1 | 1 | 1 | 1 | 1 | 1 | 1 | 1 | 1 | 1 | 1 | 1 | 1 | 1 |
| 1 | 2 | 1 | 1 | 1 | 1 | 1 | 1 | 1 | 1 | 2 | 1 | 1 | 1 |
| 1 | 1 | 1 | 1 | 1 | 1 | 1 | 1 | 1 | 1 | 1 | 1 | 1 | 1 |
| 1 | 2 | 1 | 1 | 1 | 1 | 1 | 1 | 2 | 1 | 2 | 1 | 2 | 1 |
| 1 | 1 | 2 | 1 | 1 | 1 | 1 | 1 | 1 | 1 | 1 | 1 | 1 | 1 |
| 1 | 1 | 1 | 2 | 1 | 1 | 1 | 1 | 1 | 1 | 1 | 1 | 1 | 1 |
| 1 | 1 | 1 | 1 | 1 | 1 | 1 | 1 | 1 | 1 | 1 | 1 | 1 | 1 |
| 1 | 1 | 1 | 1 | 1 | 1 | 1 | 1 | 1 | 1 | 1 | 1 | 1 | 1 |
| 2 | 1 | 1 | 1 | 1 | 1 | 1 | 1 | 2 | 2 | 1 | 1 | 1 | 1 |
| 1 | 2 | 1 | 1 | 2 | 1 | 1 | 1 | 1 | 1 | 1 | 1 | 1 | 1 |
| 1 | 1 | 1 | 1 | 1 | 1 | 1 | 1 | 1 | 1 | 1 | 1 | 2 | 1 |
| 1 | 1 | 1 | 1 | 1 | 1 | 1 | 1 | 1 | 1 | 1 | 1 | 1 | 1 |
| 1 | 1 | 1 | 1 | 1 | 1 | 1 | 1 | 2 | 1 | 1 | 1 | 1 | 1 |
| 1 | 1 | 1 | 1 | 1 | 1 | 1 | 1 | 1 | 1 | 1 | 1 | 1 | 1 |
| 1 | 1 | 1 | 2 | 1 | 1 | 1 | 1 | 1 | 1 | 1 | 1 | 1 | 1 |
| 1 | 1 | 1 | 1 | 1 | 1 | 1 | 1 | 1 | 1 | 1 | 1 | 1 | 1 |
| 1 | 1 | 1 | 1 | 1 | 1 | 2 | 1 | 1 | 1 | 1 | 1 | 1 | 1 |
| 1 | 1 | 1 | 2 | 1 | 1 | 1 | 1 | 1 | 1 | 1 | 1 | 1 | 1 |

| | | | | | | | | | | | | | |
|---|---|---|---|---|---|---|---|---|---|---|---|---|---|
| 2 | 2 | 1 | 1 | 1 | 1 | 1 | 1 | 1 | 1 | 2 | 1 | 1 | 1 |
| 1 | 1 | 1 | 2 | 1 | 1 | 1 | 1 | 1 | 1 | 2 | 1 | 1 | 1 |
| 1 | 1 | 1 | 1 | 1 | 1 | 1 | 1 | 1 | 1 | 1 | 1 | 1 | 1 |
| 1 | 1 | 1 | 1 | 1 | 1 | 1 | 1 | 1 | 1 | 1 | 1 | 1 | 1 |
| 1 | 1 | 1 | 1 | 1 | 1 | 1 | 1 | 1 | 1 | 1 | 1 | 1 | 1 |
| 1 | 1 | 1 | 1 | 1 | 1 | 1 | 1 | 1 | 1 | 1 | 1 | 1 | 1 |
| 1 | 1 | 1 | 1 | 1 | 1 | 1 | 1 | 1 | 1 | 1 | 1 | 1 | 1 |
| 1 | 1 | 1 | 1 | 1 | 1 | 1 | 1 | 1 | 1 | 2 | 1 | 1 | 1 |
| 1 | 1 | 1 | 1 | 1 | 1 | 1 | 1 | 1 | 1 | 1 | 1 | 1 | 1 |
| 1 | 1 | 1 | 1 | 1 | 1 | 1 | 1 | 1 | 1 | 1 | 1 | 1 | 1 |
| 1 | 1 | 2 | 1 | 1 | 1 | 1 | 1 | 2 | 2 | 1 | 1 | 1 | 1 |
| 1 | 1 | 1 | 1 | 1 | 1 | 1 | 1 | 1 | 1 | 1 | 1 | 1 | 1 |
| 1 | 1 | 1 | 1 | 1 | 1 | 1 | 1 | 1 | 1 | 1 | 1 | 1 | 1 |
| 1 | 2 | 1 | 1 | 2 | 1 | 1 | 1 | 2 | 2 | 1 | 1 | 1 | 1 |
| 1 | 2 | 1 | 1 | 1 | 1 | 1 | 1 | 1 | 1 | 2 | 1 | 1 | 1 |
| 1 | 1 | 1 | 1 | 1 | 1 | 1 | 1 | 1 | 1 | 1 | 1 | 1 | 1 |
| 1 | 1 | 1 | 1 | 1 | 1 | 1 | 1 | 1 | 1 | 1 | 1 | 2 | 1 |
| 2 | 2 | 1 | 1 | 1 | 1 | 1 | 1 | 2 | 1 | 1 | 1 | 1 | 1 |
| 1 | 1 | 2 | 1 | 1 | 1 | 1 | 1 | 1 | 1 | 1 | 1 | 1 | 1 |
| 1 | 1 | 1 | 1 | 1 | 1 | 1 | 1 | 1 | 1 | 1 | 1 | 1 | 1 |
| 2 | 1 | 1 | 1 | 2 | 1 | 1 | 1 | 2 | 2 | 1 | 1 | 1 | 1 |
| 1 | 1 | 2 | 1 | 2 | 1 | 1 | 1 | 1 | 1 | 1 | 1 | 1 | 1 |
| 1 | 1 | 2 | 1 | 2 | 1 | 1 | 1 | 1 | 1 | 1 | 1 | 1 | 1 |
| 1 | 1 | 1 | 1 | 1 | 1 | 1 | 1 | 1 | 1 | 1 | 1 | 1 | 1 |
| 1 | 1 | 1 | 1 | 1 | 1 | 1 | 1 | 1 | 1 | 1 | 1 | 1 | 1 |
| 1 | 1 | 1 | 1 | 1 | 1 | 1 | 1 | 1 | 1 | 1 | 1 | 1 | 1 |
| 1 | 1 | 1 | 1 | 1 | 1 | 1 | 1 | 1 | 1 | 1 | 1 | 1 | 1 |
| 1 | 2 | 1 | 2 | 1 | 2 | 1 | 1 | 2 | 1 | 1 | 1 | 1 | 1 |
| 1 | 1 | 1 | 1 | 1 | 1 | 1 | 1 | 1 | 1 | 1 | 1 | 1 | 1 |
| 1 | 1 | 1 | 1 | 1 | 1 | 1 | 1 | 2 | 1 | 1 | 1 | 1 | 1 |

| linfoma | metastas | aids | preuti | posuti | sedado | trauma | vni | vm | infec | sepse | cradmiss | cruti | crultm |
|---|---|---|---|---|---|---|---|---|---|---|---|---|---|
| 1 | 1 | 1 | 2 | 3 | 2 | 1 | 1 | 2 | 1 | 1 | 0,7 | 1,1 | 1,1 |
| 1 | 1 | 1 | 1 | 2 | 1 | 1 | 2 | 1 | 1 | 1 | 0,6 | 0,4 | 0,4 |
| 1 | 1 | 1 | 2 | 33 | 1 | 1 | 1 | 2 | 1 | 1 | 0,7 | 0,7 | 0,6 |
| 1 | 1 | 1 | 1 | 13 | 2 | 2 | 1 | 2 | 2 | 1 | 1,3 | 0,8 | 0,7 |
| 1 | 1 | 1 | 1 | 13 | 1 | 2 | 1 | 2 | 2 | 1 | 1 | 0,8 | 0,7 |
| 1 | 1 | 1 | 1 | 33 | 2 | 1 | 1 | 2 | 2 | 1 | 1 | 2,8 | 1,1 |
| 1 | 1 | 1 | 1 | 6 | 1 | 2 | 2 | 1 | 2 | 1 | 1,1 | 1,9 | 1,7 |
| 1 | 1 | 1 | 1 | 5 | 2 | 2 | 1 | 2 | 1 | 1 | 2 | 1,2 | 0,8 |
| 1 | 1 | 1 | 6 | 4 | 2 | 1 | 1 | 2 | 2 | 2 | 1,3 | 0,8 | 0,7 |
| 1 | 1 | 1 | 1 | 2 | 1 | 1 | 1 | 1 | 1 | 1 | 0,5 | 0,5 | 0,5 |
| 1 | 1 | 1 | 3 | 16 | 2 | 1 | 1 | 2 | 2 | 1 | 1,3 | 0,8 | 0,7 |
| 1 | 1 | 1 | 1 | 7 | 2 | 1 | 1 | 2 | 1 | 1 | 0,5 | 0,5 | 0,5 |
| 1 | 1 | 1 | 1 | 3 | 1 | 2 | 1 | 2 | 1 | 1 | 1,3 | 0,9 | 0,7 |
| 1 | 1 | 1 | 3 | 2 | 2 | 1 | 1 | 2 | 2 | 2 | 0,4 | 0,5 | 0,5 |
| 1 | 1 | 1 | 1 | 23 | 2 | 1 | 1 | 2 | 2 | 1 | 1,2 | 1,7 | 1,7 |
| 1 | 1 | 1 | 7 | 30 | 1 | 1 | 2 | 1 | 2 | 1 | 1,5 | 5,5 | 3,5 |
| 1 | 1 | 1 | 1 | 5 | 2 | 2 | 1 | 2 | 1 | 1 | 1 | 0,9 | 0,8 |
| 1 | 1 | 1 | 3 | 11 | 2 | 1 | 1 | 2 | 1 | 1 | 0,6 | 0,6 | 0,5 |
| 1 | 1 | 1 | 1 | 7 | 2 | 1 | 1 | 2 | 1 | 1 | 0,8 | 0,6 | 0,4 |
| 1 | 1 | 1 | 1 | 14 | 2 | 2 | 1 | 2 | 1 | 1 | 0,6 | 0,6 | 0,6 |
| 1 | 1 | 1 | 3 | 11 | 2 | 1 | 1 | 2 | 2 | 2 | 1,7 | 1 | 0,6 |
| 1 | 1 | 1 | 1 | 2 | 1 | 2 | 1 | 2 | 1 | 1 | 0,6 | 0,7 | 0,7 |
| 1 | 1 | 1 | 1 | 26 | 2 | 2 | 1 | 2 | 2 | 1 | 0,8 | 0,7 | 0,5 |
| 1 | 1 | 1 | 1 | 13 | 1 | 1 | 1 | 2 | 1 | 1 | 1,3 | 1,2 | 1 |
| 1 | 1 | 1 | 1 | 19 | 1 | 1 | 1 | 2 | 2 | 1 | 0,4 | 0,4 | 0,3 |
| 1 | 1 | 1 | 1 | 1 | 1 | 2 | 1 | 1 | 1 | 1 | 0,5 | 0,4 | 0,4 |
| 1 | 1 | 1 | 1 | 2 | 2 | 1 | 1 | 2 | 1 | 1 | 0,5 | 0,7 | 0,7 |
| 1 | 1 | 1 | 1 | 3 | 1 | 1 | 1 | 2 | 2 | 2 | 0,4 | 0,3 | 0,3 |
| 1 | 1 | 1 | 7 | 7 | 1 | 1 | 1 | 2 | 2 | 1 | 0,7 | 0,7 | 0,5 |
| 1 | 1 | 1 | 1 | 17 | 2 | 1 | 1 | 2 | 1 | 1 | 0,6 | 0,7 | 0,5 |

| | | | | | | | | | | | | | |
|---|---|---|---|---|---|---|---|---|---|---|---|---|---|
| 1 | 1 | 1 | 5 | 7 | 1 | 1 | 2 | 1 | 1 | 1 | 1,2 | 1,1 | 1 |
| 1 | 1 | 1 | 1 | 2 | 2 | 1 | 1 | 2 | 1 | 1 | 0,9 | 0,5 | 0,3 |
| 1 | 1 | 1 | 1 | 3 | 1 | 2 | 1 | 2 | 1 | 1 | 0,9 | 0,9 | 0,9 |
| 1 | 1 | 1 | 4 | 1 | 1 | 1 | 1 | 1 | 1 | 1 | 1,1 | 1 | 1 |
| 1 | 1 | 1 | 1 | 5 | 1 | 1 | 1 | 2 | 1 | 1 | 0,6 | 0,7 | 0,6 |
| 1 | 1 | 1 | 1 | 1 | 1 | 1 | 1 | 1 | 2 | 1 | 0,7 | 0,6 | 0,6 |
| 1 | 1 | 1 | 6 | 6 | 1 | 2 | 1 | 1 | 1 | 1 | 0,3 | 0,5 | 0,4 |
| 1 | 1 | 1 | 1 | 5 | 1 | 1 | 1 | 1 | 1 | 1 | 1,5 | 3,6 | 3,6 |
| 1 | 1 | 1 | 1 | 27 | 1 | 1 | 1 | 2 | 2 | 2 | 1,7 | 0,9 | 0,4 |
| 1 | 1 | 1 | 1 | 3 | 1 | 2 | 2 | 1 | 1 | 1 | 0,7 | 0,6 | 0,6 |
| 1 | 1 | 1 | 1 | 6 | 2 | 1 | 1 | 2 | 1 | 1 | 0,7 | 0,7 | 0,7 |
| 1 | 1 | 1 | 1 | 26 | 2 | 2 | 1 | 2 | 1 | 1 | 1,4 | 1,1 | 0,9 |
| 1 | 1 | 1 | 3 | 5 | 1 | 1 | 2 | 1 | 1 | 1 | 0,6 | 0,8 | 0,6 |
| 1 | 1 | 1 | 1 | 2 | 1 | 1 | 1 | 1 | 1 | 1 | 0,7 | 0,6 | 0,6 |
| 1 | 1 | 1 | 1 | 5 | 1 | 1 | 2 | 1 | 1 | 1 | 0,5 | 0,6 | 0,5 |
| 1 | 1 | 1 | 6 | 5 | 1 | 1 | 2 | 1 | 1 | 1 | 0,9 | 1 | 1 |
| 1 | 1 | 1 | 6 | 4 | 1 | 2 | 2 | 1 | 1 | 1 | 0,7 | 0,8 | 0,8 |
| 1 | 1 | 1 | 1 | 2 | 1 | 2 | 2 | 1 | 2 | 1 | 0,7 | 0,9 | 0,9 |
| 1 | 1 | 1 | 1 | 74 | 2 | 1 | 1 | 2 | 2 | 1 | 0,8 | 1,4 | 0,8 |
| 1 | 1 | 1 | 1 | 3 | 2 | 1 | 2 | 1 | 1 | 1 | 0,5 | 0,4 | 0,3 |
| 1 | 1 | 2 | 1 | 7 | 2 | 2 | 1 | 2 | 1 | 1 | 1 | 1 | 0,4 |
| 1 | 1 | 1 | 1 | 12 | 2 | 1 | 1 | 2 | 2 | 2 | 1,4 | 4,1 | 3,7 |
| 1 | 1 | 1 | 1 | 18 | 2 | 2 | 1 | 2 | 2 | 1 | 1,1 | 2,5 | 1 |
| 1 | 1 | 1 | 1 | 26 | 2 | 2 | 1 | 2 | 1 | 1 | 1 | 1 | 0,6 |
| 1 | 1 | 1 | 5 | 2 | 2 | 1 | 1 | 2 | 1 | 1 | 0,7 | 0,6 | 0,6 |
| 1 | 1 | 1 | 2 | 17 | 2 | 2 | 1 | 2 | 1 | 1 | 0,9 | 1,2 | 1,2 |
| 1 | 1 | 1 | 3 | 10 | 2 | 2 | 1 | 2 | 1 | 2 | 0,7 | 0,9 | 0,9 |
| 1 | 1 | 1 | 3 | 2 | 2 | 1 | 2 | 1 | 1 | 1 | 0,9 | 0,8 | 0,7 |
| 1 | 1 | 1 | 6 | 2 | 1 | 2 | 1 | 1 | 1 | 1 | 0,7 | 1 | 0,7 |
| 1 | 1 | 1 | 6 | 5 | 1 | 1 | 2 | 1 | 2 | 1 | 0,7 | 1,6 | 0,5 |
| 1 | 1 | 1 | 1 | 7 | 2 | 2 | 1 | 2 | 1 | 1 | 1 | 0,9 | 0,7 |

| | | | | | | | | | | | | | |
|---|---|---|---|---|---|---|---|---|---|---|---|---|---|
| 1 | 1 | 1 | 1 | 5 | 1 | 2 | 2 | 1 | 1 | 1 | 0,6 | 0,5 | 0,4 |
| 1 | 1 | 1 | 1 | 20 | 2 | 2 | 1 | 2 | 1 | 1 | 0,7 | 0,7 | 0,6 |
| 1 | 1 | 1 | 1 | 3 | 1 | 1 | 2 | 1 | 2 | 2 | 0,4 | 0,4 | 0,3 |
| 1 | 1 | 1 | 1 | 16 | 2 | 2 | 1 | 2 | 2 | 2 | 0,9 | 0,9 | 0,6 |
| 1 | 1 | 1 | 1 | 7 | 2 | 1 | 1 | 2 | 1 | 1 | 0,4 | 0,7 | 0,3 |
| 1 | 1 | 1 | 7 | 2 | 1 | 1 | 2 | 1 | 2 | 1 | 0,8 | 0,9 | 0,8 |
| 1 | 1 | 1 | 2 | 13 | 2 | 1 | 1 | 2 | 2 | 2 | 1,1 | 4,4 | 2,5 |
| 1 | 1 | 1 | 1 | 5 | 1 | 1 | 2 | 1 | 2 | 2 | 0,5 | 0,5 | 0,5 |
| 1 | 1 | 1 | 1 | 66 | 2 | 2 | 1 | 2 | 1 | 1 | 1 | 1,1 | 0,3 |
| 1 | 1 | 1 | 1 | 10 | 2 | 1 | 1 | 2 | 2 | 1 | 1,2 | 5,6 | 3,3 |
| 1 | 1 | 1 | 3 | 25 | 2 | 2 | 1 | 2 | 1 | 1 | 1,1 | 1,6 | 1,1 |
| 1 | 1 | 1 | 1 | 6 | 1 | 2 | 1 | 1 | 1 | 1 | 0,9 | 1,1 | 0,3 |
| 1 | 1 | 1 | 1 | 5 | 2 | 2 | 1 | 2 | 1 | 1 | 0,7 | 0,9 | 0,6 |
| 1 | 1 | 1 | 1 | 3 | 2 | 2 | 1 | 2 | 1 | 1 | 0,7 | 0,6 | 0,4 |
| 1 | 1 | 1 | 3 | 9 | 2 | 1 | 1 | 2 | 2 | 2 | 0,8 | 1,2 | 0,9 |
| 1 | 1 | 1 | 1 | 5 | 2 | 2 | 1 | 2 | 1 | 1 | 1,4 | 1,2 | 0,8 |
| 1 | 1 | 1 | 4 | 3 | 1 | 1 | 1 | 1 | 2 | 1 | 1,1 | 0,9 | 0,8 |
| 1 | 1 | 1 | 1 | 28 | 2 | 2 | 1 | 2 | 1 | 1 | 1,1 | 1,2 | 0,5 |
| 1 | 1 | 1 | 1 | 7 | 2 | 1 | 1 | 2 | 2 | 1 | 0,9 | 1,3 | 0,7 |
| 1 | 1 | 1 | 1 | 24 | 2 | 2 | 1 | 2 | 1 | 1 | 0,7 | 1 | 0,6 |
| 1 | 1 | 1 | 5 | 4 | 1 | 1 | 1 | 2 | 2 | 2 | 1,1 | 0,9 | 0,9 |
| 1 | 1 | 1 | 1 | 3 | 2 | 1 | 1 | 1 | 2 | 1 | 0,8 | 0,7 | 0,5 |
| 1 | 1 | 1 | 1 | 10 | 2 | 1 | 1 | 2 | 1 | 1 | 0,8 | 0,9 | 0,6 |
| 1 | 1 | 1 | 6 | 7 | 1 | 2 | 2 | 1 | 2 | 2 | 0,6 | 0,6 | 0,5 |
| 1 | 1 | 1 | 4 | 8 | 2 | 1 | 1 | 2 | 2 | 1 | 0,9 | 0,8 | 0,8 |
| 1 | 1 | 1 | 1 | 44 | 2 | 1 | 1 | 2 | 2 | 2 | 1,5 | 2,4 | 2 |
| 1 | 1 | 1 | 2 | 7 | 2 | 1 | 1 | 2 | 2 | 2 | 0,9 | 1,7 | 1,3 |
| 1 | 1 | 1 | 1 | 15 | 2 | 2 | 1 | 2 | 1 | 1 | 0,6 | 0,8 | 0,6 |
| 1 | 1 | 1 | 5 | 3 | 1 | 1 | 1 | 2 | 2 | 1 | 0,7 | 0,5 | 0,5 |
| 1 | 1 | 1 | 1 | 6 | 1 | 1 | 1 | 2 | 1 | 1 | 0,9 | 4,7 | 2,3 |
| 1 | 1 | 1 | 1 | 2 | 1 | 1 | 2 | 1 | 2 | 1 | 0,5 | 0,5 | 0,5 |

| | | | | | | | | | | | | | |
|---|---|---|---|---|---|---|---|---|---|---|---|---|---|
| 1 | 1 | 1 | 2 | 10 | 2 | 1 | 1 | 2 | 1 | 1 | 0,6 | 0,4 | 0,3 |
| 1 | 1 | 1 | 6 | 11 | 1 | 1 | 1 | 2 | 2 | 1 | 0,2 | 0,8 | 0,8 |
| 1 | 1 | 1 | 1 | 5 | 2 | 2 | 1 | 2 | 1 | 1 | 0,8 | 0,7 | 0,6 |
| 1 | 1 | 1 | 1 | 24 | 2 | 1 | 1 | 2 | 2 | 1 | 0,9 | 1,8 | 1,6 |
| 1 | 1 | 1 | 1 | 6 | 2 | 2 | 1 | 2 | 1 | 1 | 1,1 | 1,6 | 0,6 |
| 1 | 1 | 1 | 1 | 4 | 2 | 2 | 1 | 2 | 1 | 1 | 0,8 | 1 | 0,6 |
| 1 | 1 | 1 | 1 | 17 | 2 | 1 | 1 | 2 | 1 | 1 | 0,8 | 0,6 | 0,5 |
| 1 | 1 | 1 | 1 | 5 | 2 | 1 | 1 | 2 | 2 | 2 | 1,7 | 4,1 | 4,1 |
| 1 | 1 | 1 | 1 | 6 | 2 | 2 | 1 | 2 | 1 | 1 | 0,9 | 0,8 | 0,7 |
| 1 | 1 | 1 | 1 | 8 | 2 | 2 | 1 | 2 | 2 | 1 | 0,9 | 1,2 | 1 |
| 1 | 1 | 1 | 1 | 5 | 1 | 2 | 2 | 1 | 2 | 1 | 0,5 | 0,4 | 0,4 |
| 1 | 1 | 1 | 1 | 2 | 1 | 1 | 2 | 1 | 1 | 1 | 0,5 | 0,6 | 0,6 |
| 1 | 1 | 1 | 1 | 4 | 1 | 1 | 2 | 1 | 2 | 1 | 0,3 | 0,4 | 0,4 |
| 1 | 1 | 1 | 1 | 36 | 2 | 1 | 1 | 2 | 1 | 1 | 0,7 | 1,1 | 0,9 |
| 1 | 1 | 1 | 6 | 5 | 2 | 1 | 1 | 2 | 2 | 2 | 0,9 | 8,5 | 6 |
| 1 | 1 | 1 | 1 | 21 | 1 | 1 | 1 | 2 | 1 | 1 | 1,2 | 1,2 | 0,7 |
| 1 | 1 | 1 | 1 | 1 | 1 | 1 | 2 | 1 | 1 | 1 | 1,5 | 1,1 | 1,1 |
| 1 | 1 | 1 | 1 | 9 | 1 | 1 | 2 | 1 | 1 | 1 | 1,1 | 1,1 | 0,8 |
| 1 | 1 | 1 | 1 | 2 | 1 | 2 | 2 | 1 | 1 | 1 | 0,8 | 0,7 | 0,7 |
| 1 | 1 | 1 | 1 | 2 | 1 | 1 | 2 | 1 | 1 | 1 | 0,6 | 0,8 | 0,8 |
| 1 | 1 | 1 | 2 | 5 | 1 | 1 | 2 | 1 | 2 | 2 | 0,6 | 0,7 | 0,6 |
| 1 | 1 | 1 | 5 | 4 | 1 | 2 | 2 | 1 | 1 | 1 | 0,4 | 0,4 | 0,4 |
| 1 | 1 | 1 | 6 | 38 | 2 | 1 | 1 | 2 | 2 | 2 | 0,7 | 2,2 | 1,2 |
| 1 | 1 | 1 | 1 | 4 | 2 | 1 | 1 | 2 | 2 | 1 | 0,9 | 1,1 | 1,1 |
| 1 | 1 | 1 | 1 | 21 | 2 | 2 | 1 | 2 | 1 | 1 | 0,8 | 0,8 | 0,5 |
| 1 | 1 | 1 | 1 | 32 | 2 | 2 | 1 | 2 | 1 | 1 | 0,9 | 0,8 | 0,6 |
| 1 | 1 | 1 | 1 | 24 | 2 | 1 | 1 | 2 | 1 | 1 | 0,8 | 0,8 | 0,5 |
| 1 | 1 | 1 | 5 | 26 | 1 | 1 | 1 | 2 | 2 | 1 | 0,9 | 0,7 | 0,4 |
| 1 | 1 | 1 | 1 | 2 | 1 | 1 | 2 | 1 | 1 | 1 | 0,6 | 0,6 | 0,6 |
| 1 | 1 | 1 | 3 | 7 | 1 | 2 | 1 | 2 | 1 | 1 | 0,5 | 0,7 | 0,5 |

| diurese | lra | trs | desfecho | altalra |
|---|---|---|---|---|
| 1 | 2 | 1 | 2 | 1 |
| 1 | 1 | 3 | 1 | 1 |
| 4 | 1 | 3 | 1 | 1 |
| 1 | 1 | 3 | 1 | 1 |
| 1 | 1 | 3 | 1 | 1 |
| 1 | 2 | 2 | 1 | 3 |
| 1 | 2 | 1 | 1 | 4 |
| 1 | 1 | 3 | 1 | 1 |
| 1 | 1 | 3 | 1 | 1 |
| 1 | 1 | 3 | 1 | 1 |
| 1 | 1 | 3 | 1 | 1 |
| 1 | 1 | 3 | 1 | 1 |
| 1 | 1 | 3 | 2 | 1 |
| 1 | 1 | 3 | 2 | 1 |
| 1 | 2 | 1 | 1 | 4 |
| 2 | 2 | 2 | 1 | 4 |
| 1 | 1 | 3 | 1 | 1 |
| 4 | 1 | 3 | 1 | 1 |
| 1 | 2 | 1 | 1 | 2 |
| 1 | 1 | 3 | 1 | 1 |
| 1 | 1 | 3 | 1 | 1 |
| 1 | 1 | 3 | 1 | 1 |
| 1 | 1 | 3 | 1 | 1 |
| 1 | 1 | 3 | 1 | 1 |
| 1 | 1 | 3 | 1 | 1 |
| 1 | 1 | 3 | 1 | 1 |
| 1 | 1 | 3 | 1 | 1 |
| 1 | 1 | 3 | 1 | 1 |
| 1 | 1 | 3 | 1 | 1 |
| 1 | 1 | 3 | 1 | 1 |

| | | | | |
|---|---|---|---|---|
| 1 | 1 | 3 | 1 | 1 |
| 1 | 1 | 3 | 2 | 1 |
| 1 | 1 | 3 | 1 | 1 |
| 1 | 1 | 3 | 1 | 1 |
| 4 | 1 | 3 | 1 | 1 |
| 1 | 1 | 3 | 1 | 1 |
| 1 | 1 | 3 | 1 | 1 |
| 1 | 2 | 1 | 2 | 1 |
| 1 | 1 | 3 | 1 | 1 |
| 1 | 1 | 3 | 1 | 1 |
| 1 | 1 | 3 | 1 | 1 |
| 1 | 1 | 3 | 1 | 1 |
| 1 | 1 | 3 | 1 | 1 |
| 1 | 1 | 3 | 1 | 1 |
| 1 | 1 | 3 | 1 | 1 |
| 1 | 1 | 3 | 1 | 1 |
| 1 | 1 | 3 | 1 | 1 |
| 1 | 1 | 3 | 1 | 1 |
| 1 | 2 | 1 | 1 | 2 |
| 1 | 1 | 3 | 1 | 1 |
| 1 | 1 | 3 | 1 | 1 |
| 1 | 2 | 2 | 1 | 4 |
| 1 | 2 | 1 | 1 | 2 |
| 1 | 1 | 3 | 1 | 1 |
| 1 | 1 | 3 | 1 | 1 |
| 1 | 1 | 3 | 1 | 1 |
| 1 | 1 | 3 | 2 | 1 |
| 1 | 1 | 3 | 1 | 1 |
| 3 | 2 | 1 | 1 | 2 |
| 4 | 2 | 1 | 1 | 2 |
| 1 | 1 | 3 | 1 | 1 |

| | | | | |
|---|---|---|---|---|
| 1 | 1 | 3 | 1 | 1 |
| 1 | 1 | 3 | 1 | 1 |
| 1 | 1 | 3 | 1 | 1 |
| 1 | 1 | 3 | 1 | 1 |
| 1 | 2 | 1 | 1 | 2 |
| 1 | 1 | 3 | 1 | 1 |
| 1 | 2 | 2 | 1 | 4 |
| 1 | 1 | 3 | 1 | 1 |
| 1 | 2 | 1 | 1 | 2 |
| 1 | 2 | 2 | 2 | 1 |
| 1 | 2 | 1 | 1 | 2 |
| 3 | 1 | 3 | 1 | 1 |
| 1 | 1 | 3 | 1 | 1 |
| 3 | 1 | 3 | 1 | 1 |
| 1 | 2 | 1 | 1 | 3 |
| 1 | 1 | 3 | 1 | 1 |
| 1 | 1 | 3 | 1 | 1 |
| 1 | 1 | 3 | 1 | 1 |
| 1 | 2 | 1 | 1 | 2 |
| 1 | 1 | 3 | 1 | 1 |
| 1 | 1 | 3 | 1 | 1 |
| 1 | 1 | 3 | 1 | 1 |
| 1 | 1 | 3 | 1 | 1 |
| 1 | 1 | 3 | 1 | 1 |
| 1 | 1 | 3 | 1 | 1 |
| 1 | 2 | 1 | 1 | 3 |
| 1 | 2 | 2 | 2 | 1 |
| 1 | 1 | 3 | 1 | 1 |
| 1 | 1 | 3 | 1 | 1 |
| 1 | 2 | 2 | 1 | 4 |
| 3 | 1 | 3 | 1 | 1 |

| | | | | |
|---|---|---|---|---|
| 1 | 1 | 3 | 1 | 1 |
| 4 | 2 | 1 | 1 | 4 |
| 1 | 1 | 3 | 1 | 1 |
| 1 | 2 | 1 | 2 | 1 |
| 1 | 2 | 1 | 1 | 2 |
| 1 | 1 | 3 | 1 | 1 |
| 1 | 1 | 3 | 1 | 1 |
| 1 | 2 | 2 | 2 | 1 |
| 1 | 1 | 3 | 1 | 1 |
| 1 | 2 | 1 | 1 | 3 |
| 1 | 1 | 3 | 1 | 1 |
| 1 | 1 | 3 | 1 | 1 |
| 1 | 1 | 3 | 1 | 1 |
| 4 | 1 | 3 | 1 | 1 |
| 1 | 2 | 2 | 2 | 1 |
| 1 | 1 | 3 | 1 | 1 |
| 1 | 1 | 3 | 1 | 1 |
| 1 | 1 | 3 | 1 | 1 |
| 1 | 1 | 3 | 1 | 1 |
| 1 | 1 | 3 | 1 | 1 |
| 1 | 1 | 3 | 1 | 1 |
| 4 | 1 | 3 | 1 | 1 |
| 1 | 2 | 2 | 1 | 3 |
| 1 | 1 | 3 | 1 | 1 |
| 1 | 1 | 3 | 1 | 1 |
| 1 | 1 | 3 | 1 | 1 |
| 1 | 1 | 3 | 1 | 1 |
| 1 | 1 | 3 | 1 | 1 |
| 1 | 1 | 3 | 1 | 1 |
| 1 | 1 | 3 | 1 | 1 |

| | |
|---|---|
| ID | Patient number |
| codpesq | Patient code |
| idade | Age |
| sexo | Sex |
| corpele | Self-declaration of skin color |
| setorig | Hospital department in which the patient has been originally admitted |
| histetil | History of daily alcohol consumption |
| tabagis | Smoking status |
| apache_ii | Acute Physiology and Chronic Health Evaluation II (APACHE II) |
| cid10 | International Classification of Diseases for Mortality and Morbidity Statistics (ICD-10) |
| comorb | Comorbidities |
| iam | Myocardial infarction |
| icc | Congestive heart failure |
| dvp | Peripheral vascular disease |
| dcervasc | Cerebrovascular disease |
| demencia | Dementia |
| dpulmona | Chronic lung disease |
| dconjunt | Connective Tissue Disease |
| ulcera | Ulcer |
| hepleve | Liver disease |
| dhepseve | Severe liver disease |
| diabetes | Diabetes |
| diabcomp | Complications of Diabetes |
| hemipleg | Hemiplegia or paraplegia |
| drenal | Kidney disease |
| neoplas | Neoplasms |
| leucem | Leukaemia |
| linfoma | Malignant lymphoma |
| metastas | Malignant neoplasm metastases |
| aids | SIDA/HIV |
| preuti | Hospitalization time before admission to ICU |
| posuti | Hospitalization time after admission to ICU |
| sedado | Presence of sedation |
| trauma | Presence of trauma |
| vni | Non-invasive mechanical ventilation |
| vm | Mechanical ventilation |
| infec | Infection |
| sepse | Sepsis |
| cradmiss | First SCr recorded upon hospital admission |
| cruti | First SCr measurements at ICU |
| crultm | Last SCr measurements at ICU |
| diurese | Diuresis measurements |
| lra | AKI |
| trs | Renal replacement therapy |
| desfecho | Outcome (Discharge, death or transferred to other institutions) |
| altalra | Status of recovery for AKI group |
